# Can We Predict Individual Combined Benefit and Harm of Therapy? Warfarin Therapy for Atrial Fibrillation as a Test Case

**DOI:** 10.1371/journal.pone.0160713

**Published:** 2016-08-11

**Authors:** Guowei Li, Lehana Thabane, Thomas Delate, Daniel M. Witt, Mitchell A. H. Levine, Ji Cheng, Anne Holbrook

**Affiliations:** 1 Department of Clinical Epidemiology & Biostatistics, McMaster University, Hamilton, ON, Canada; 2 St. Joseph's Hospital, McMaster University, Hamilton, ON, Canada; 3 Kaiser Permanente Colorado Clinical Pharmacy Research Team, Aurora, CO, United States of America; 4 University of Colorado Skaggs School of Pharmacy and Pharmaceutical Sciences, Denver, CO, United States of America; 5 Department of Pharmacotherapy, University of Utah, Salt Lake City, Utah, United States of America; 6 Division of Clinical Pharmacology & Toxicology, Department of Medicine, McMaster University, Hamilton, ON, Canada; Fraunhofer Research Institution of Marine Biotechnology, GERMANY

## Abstract

**Objectives:**

To construct and validate a prediction model for individual combined benefit and harm outcomes (stroke with no major bleeding, major bleeding with no stroke, neither event, or both) in patients with atrial fibrillation (AF) with and without warfarin therapy.

**Methods:**

Using the Kaiser Permanente Colorado databases, we included patients newly diagnosed with AF between January 1, 2005 and December 31, 2012 for model construction and validation. The primary outcome was a prediction model of composite of stroke or major bleeding using polytomous logistic regression (PLR) modelling. The secondary outcome was a prediction model of all-cause mortality using the Cox regression modelling.

**Results:**

We included 9074 patients with 4537 and 4537 warfarin users and non-users, respectively. In the derivation cohort (n = 4632), there were 136 strokes (2.94%), 280 major bleedings (6.04%) and 1194 deaths (25.78%) occurred. In the prediction models, warfarin use was not significantly associated with risk of stroke, but increased the risk of major bleeding and decreased the risk of death. Both the PLR and Cox models were robust, internally and externally validated, and with acceptable model performances.

**Conclusions:**

In this study, we introduce a new methodology for predicting individual combined benefit and harm outcomes associated with warfarin therapy for patients with AF. Should this approach be validated in other patient populations, it has potential advantages over existing risk stratification approaches as a patient-physician aid for shared decision-making

## Introduction

Atrial fibrillation (AF) is a common, age-related, chronic arrhythmia that is a major risk factor for stroke and mortality [1,2]. The presence of AF increases the risk of stroke five-fold independently [3], and doubles the risk of death from AF-related stroke [2]. At present, oral anticoagulants are the mainstay for stroke prophylaxis in patients with AF [4]. Despite the growth in use of newer oral anticoagulants, warfarin remains a frequently used antithrombotic therapy for AF, where it lowers rates of stroke as well as mortality [2,4–6]. However, the use of anticoagulants also is associated with an increased risk of major bleeding including intracranial hemorrhage (ICH) [5]. Thus, this combination of potential life-saving benefit and life-threatening harm may dissuade clinicians from prescribing warfarin for eligible patients [7–11].

Clinical prediction rules such as the CHADS_2_ (**C**ongestive heart failure, **H**ypertension, **A**ge > 75 years, **D**iabetes, Previous **s**troke [2 points]) and the CHA_2_DS_2_-VASc (**C**ongestive heart failure; **H**ypertension; **A**ge ≥ 75 years [2 points]; **D**iabetes mellitus; **S**troke [2 points], **V**ascular disease, **A**ge 65–74 years, and **S**ex category [female]) scores have been developed and widely used to predict stroke risk in AF patients [2,5,12,13]. Likewise, the HAS-BLED score (**H**ypertension; **A**bnormal renal/liver function; **S**troke history; **B**leeding history or predisposition; **L**abile international normalized ratio [INR], **E**lderly [>65 years]; **D**rugs/alcohol concomitantly) has been validated to predict risk of major bleeding with warfarin therapy [2,5,14–17]. Unfortunately, the CHADS_2_, CHA_2_DS_2_-VASc and HAS-BLED scores were not derived from the same patients or populations. Specifically, the CHADS_2_ used data from 1733 patients in the US National Registry of AF [13], while the CHA_2_DS_2_-VASc and HAS-BLED scores were both developed from the Euro Heart Survey on AF population but used data on 1084 and 3978 patients respectively [12,14]. Thus these scores are unable to assess simultaneously a patient’s potential for benefit and/or harm with warfarin therapy, yet this is exactly what each patient wants to know [18].

While the CHADS_2_, CHA_2_DS_2_-VASc and HAS-BLED scores help estimate an individual’s chance of benefit and harm separately, a more sophisticated methodology is needed. The ‘net benefit’ approach involves calculating the main benefit of warfarin therapy (reduced risk of stroke or systemic embolism) then deducting the main harm (weight*increased risk of ICH, weight = 1.5) in the same population [19–21]. However, this approach does not take into account gastrointestinal (GI) bleeding risk, and the weighting for ICH is chosen arbitrarily.

In general, treatment effects of warfarin therapy for individual patients can be divided into four quadrants: 1) benefit without harm; 2) harm without benefit; 3) neither benefit or harm; and 4) both benefit and harm simultaneously (**Table 1**). A method for predicting the probabilities of the four outcome quadrants (i.e., individualized combined benefit and harm outcomes) for each patient is needed. The polytomous logistic regression (PLR) modelling can be used for predictions due to the four multinomial levels of outcomes [22,23]. Therefore, the objective of this study was to use the PLR modelling to construct and externally validate a prediction model for patients’ individual combined benefit and harm outcomes (stroke with no major bleeding, major bleeding with no stroke, neither event, or both stroke and major bleeding) with and without warfarin therapy for AF. In real-world clinical settings, the prediction of individualized combined benefit and harm outcomes related to warfarin therapy could assist with the patient-physician shared decision-making process.

**Table 1:** Warfarin’s combined benefit and harm outcomes.

	Harm (major bleeding)	No harm (no major bleeding)
**Benefit (no stroke)**	No stroke/major bleeding	No stroke/no major bleeding
**No benefit (stroke)**	Stroke/major bleeding	Stroke/no major bleeding

## Methods

### Study design and setting

The methods have been described in detail previously [18]. Briefly, Kaiser Permanente Colorado (KPCO), a non-profit, integrated health care delivery system in the U.S. Denver-Boulder metropolitan area, utilizes a centralized anticoagulation service that provides anticoagulation services for KPCO patients with AF [24,25]. KPCO maintains extensive medical, pharmacy, laboratory, utilization, mortality, and membership electronic, integrated administrative datasets. Data were extracted for KPCO patients diagnosed with AF who were and were not prescribed warfarin therapy and analyzed at St. Joseph’s Healthcare Hamilton in Hamilton, ON. The KPCO Institutional Review Board and the Hamilton Integrated Research Ethics Board approved this study with a waiver for informed consent.

Patients newly diagnosed with AF between January 1, 2005 and December 31, 2012 were included. Newly diagnosed status was defined by absence of AF diagnosis in the previous 180 days. Patients were followed for up to 180 days after AF diagnosis to assess if warfarin therapy was initiated. Patients who had at least one warfarin purchase or no warfarin purchases were grouped as warfarin *users* and *non-users*, respectively. Warfarin non-users were randomly matched 1:1 to warfarin users on year of AF diagnosis [26]. Patients with AF diagnosed between January 1, 2005 and December 31, 2008 comprised the derivation cohort (KPCO-I), while patients with AF diagnosed between January 1, 2009 and December 31, 2012 comprised the validation cohort (KPCO-II). Compared with internal validation by randomly splitting the entire dataset, separating derivation and validation cohorts by AF diagnosis dates enabled a external validation of the model independent of the original data and development process [27]. In addition, the separation by dates of AF diagnoses could also account for changes in standards of care and management for patients over time.

### Study patients

The date of AF diagnosis for each patient was defined as study ***start date***. To include as many outcomes as possible, study ***outcome end date*** was defined as June 30, 2009 and June 30, 2013 for the derivation and validation cohorts, respectively. To control the potential of immortal time bias, the study ***index date*** for warfarin users was defined as the first warfarin purchase date after start date [28,29]. Warfarin non-users were assigned an index date corresponding to the length of time from study start date to the index date of their randomly-matched warfarin user [26]. Warfarin non-users who died prior to their assigned index date were excluded from the analyses, because they were unable to be chosen to enter the cohort [26]. Patients were followed from index date until KPCO plan disenrollment, death, or study outcome end date, whichever came first [18].

### Outcomes

The primary outcome was a prediction model of composite of stroke or major bleeding. The secondary outcome was a prediction model of all-cause death. All of the outcomes were assessed from the index date to outcome end date. For the prediction model of stroke or major bleeding, we categorized patients into one of the four outcome groups based on their survival time to first event: stroke with no major bleeding, major bleeding with no stroke, neither event, or both stroke and major bleeding. For the prediction model of all-cause mortality, patients were categorized into survival or non-survival groups.

Stroke and major bleeding events were identified during an ambulatory KPCO medical office visit, emergency department (ED) visit, or inpatient stay using International Classification of Disease, Ninth Revision, Clinical Modification (ICD-9-CM) codes in the primary position. Major bleeding was defined as bleeding that led to a hospital admission or an ED visit requiring a transfusion [30]. However, bleeding that caused a drop in hemoglobin of ≥ 20g/L but did not necessitate a transfusion [31] was not included as major bleeding since no inpatient or ED hemoglobin laboratory values were available. ICH was categorized as major bleeding, rather than stroke. Stroke or major bleeding occurring before the index date was categorized as a risk factor (i.e., prior stroke, prior major bleeding) rather than a study outcome [18].

### Potential predictors of benefit and/or harm

The potential predictors used in this study included patients’ demographic characteristics (i.e., sex, age), laboratory measures, baseline comorbidities, warfarin use, and concurrent use of medications that interact with warfarin. Laboratory measurements included INR, hemoglobin, serum creatinine and albumin recorded most proximal but prior to the index date. Comorbidities were from ambulatory KPCO medical office visits in the 180 days prior to the index date. Comorbidities were components of the CHA_2_DS_2-_VASc and HAS-BLED schemes, as well as components included in the Charlson Comorbidity Index [32]. Data on warfarin use included the length of time from study start date to the first purchase date, the length of time for each dispensed warfarin prescription from index date, and days of warfarin supplied. Concurrent use of other medications included purchases for medications made during the 90 days after index date. We included concurrent medications for which there was evidence of an interaction that potentiated or inhibited the effect of warfarin. The list of included medications was from two systematic reviews that investigated warfarin interactions with other drugs [33,34].

### Statistical analyses

All tests were two-sided with a significance level of 0.05, unless otherwise specified. We described continuous variables as means (+/- standard deviations [SDs]), and frequencies and percentages for categorical variables. Student’s t-tests were used to compare continuous variables and chi-square tests of associations were applied for categorical variables. In the derivation and validation cohort, we assessed the stroke and major bleeding incidence rate trends stratified by the CHA_2_DS_2_-VASc score and HAS-BLED score, respectively.

### Model building

PLR modeling was used to develop a prediction model for the four individual benefit and harm outcomes using the neither event group as the referent category. Odds ratios (ORs) with 95% confidence intervals (CIs) were used to quantify the relationship between outcomes and predictors. We employed Cox proportional hazards regression analysis to build a prediction model for all-cause mortality, using hazard ratios (HRs) to quantify the associations between predictors and mortality. All of the analyses were adjusted for matching of warfarin users and non-users.

Both the PLR and Cox regression models followed the same procedures for model construction. First, the effect of multicollinearity was evaluated using the criterion of a variance inflation factor ≥ 4 to prune candidate predictors. Subsequently, we performed univariate analyses to select all possible predictors with a p-value ≤ 0.20 to enter the multivariable analyses. And then the predictors with a p-value < 0.05 in the multivariable analyses were retained in the prediction models. Lastly we identified significant two-way interactions to finalize our prediction models [35].

For the primary outcome, three sensitivity analyses were performed by: 1) using multiple imputations if missing data were ≥ 10%; 2) treating the use of warfarin as a time-dependent covariate to evaluate the effect of warfarin on stroke and major bleeding, using a gap of > 30 days to indicate warfarin discontinuation [36]; and 3) employing a competing risk analysis using the Fine and Gray method to take into account all-cause mortality as a competing risk of stroke and major bleeding [37].

### Model performance and validation

Comparison between the predicted and observed risks in deciles was used to evaluate calibration of the prediction models. Discrimination was measured by the area under the receiver operating characteristic curves (AUCs) for the PLR model and Harrell's C index for the Cox model. Goodness-of-fit was assessed by a Hosmer-Lemeshow statistic [38] and Gronnesby and Borgan test [39] with ten groups based on the predicted risk scores for the PLR and Cox models, respectively.

Two internal validations were performed for the PLR model by using 10-fold cross-validation [40] and bootstrap analysis [41]. We also used bootstrap analysis to internally validate the Cox model for all-cause mortality. For the external validation, because the incidence rates of outcomes were different from the derivation and validation cohorts and there was evidence that the original models were not a good fit to the validation cohort, we updated the models’ intercepts as well as the regression coefficients by using the calibration intercepts and calibration slopes [23,42,43]. The evaluation of goodness-of-fit, calibration, and discrimination was repeated in the validation cohort.

Analyses were performed with the software packages SAS Version 9.3 (SAS Institute, Inc., Cary, NC) and STATA Version 12 (Stata Corp., College Station, TX, USA). For the calibration plots of the PLR model, we used the software R version 3.2.1 (R Foundation for Statistical Computing, Vienna, Austria) with the Design library.

## Results

### Patient characteristics

We included 9074 patients diagnosed with AF with 4537 and 4537 warfarin users and non-users, respectively (see **S1 Fig** for patient dispositions). Overall mean age was 71.7 years (SD: 13.0) and 46% were female (**Table 2**). Overall mean CHA_2_DS_2_-VASc and HAS-BLED scores were 2.99 (SD: 1.56) and 1.73 (SD: 0.88), respectively.

**Table 2:** Characteristics of study patients stratified by warfarin users versus non-users in derivation and validation cohort.

Baseline Characteristics	Total participants (n = 9074)	KPCO-I (n = 4632)^1^			KPCO-II (n = 4442)^2^		
		Warfarin users (n = 2316)	Warfarin non-users (n = 2316)	P-value	Warfarin users (n = 2221)	Warfarin non-users (n = 2221)	P-value
**Age:** mean (SD), years	71.7 (13.00)	72.3 (10.74)	70.5 (15.26)	<0.001	72.9 (10.64)	71.3 (14.54)	<0.001
**Female**: n (%)	4199 (46.28)	1229 (53.07)	1209 (52.20)	0.556	1275 (57.41)	1162 (52.32)	<0.001
**Comorbidities:** n (%)							
Congestive heart failure	1064 (11.73)	286 (12.35)	220 (9.50)	0.002	325 (14.63)	233 (10.49)	<0.001
Hypertension	7132 (78.60)	2024 (87.39)	1609 (69.47)	<0.001	1957 (88.11)	1542 (69.43)	<0.001
Diabetes	1759 (19.39)	428 (18.48)	391 (16.88)	0.154	509 (22.92)	431 (19.41)	0.004
Prior stroke/TIA	539 (5.94)	122 (5.27)	112 (4.84)	0.502	197 (8.87)	108 (4.86)	<0.001
Myocardial infarction	516 (5.69)	93 (4.02)	94 (4.06)	0.941	183 (8.24)	146 (6.57)	0.034
Peripheral vascular disease	615 (6.78)	138 (5.96)	129 (5.57)	0.571	183 (8.24)	165 (7.43)	0.315
Renal disease	1146 (12.63)	174 (7.51)	219 (9.46)	0.018	406 (18.28)	347 (15.62)	0.018
Liver disease	20 (0.22)	3 (0.13)	4 (0.17)	0.705#	2 (0.09)	11 (0.50)	0.022#
Prior major bleeding	260 (2.87)	74 (3.20)	103 (4.45)	0.026	42 (1.89)	41 (1.85)	0.912
Anemia	657 (7.24)	142 (6.13)	189 (8.16)	0.007	127 (5.72)	199 (8.96)	<0.001
Alcohol abuse	119 (1.31)	15 (0.65)	34 (1.47)	0.006	24 (1.08)	46 (2.07)	0.008
Other cerebrovascular disease	194 (2.14)	38 (1.64)	43 (1.86)	0.575	58 (2.61)	55 (2.48)	0.775
Dementia	21 (0.23)	2 (0.09)	4 (0.17)	0.687#	1 (0.05)	14 (0.63)	<0.001#
Chronic pulmonary disease	468 (5.16)	115 (4.97)	89 (3.84)	0.063	153 (6.89)	111 (5.00)	0.008
Rheumatic disease	245 (2.70)	67 (2.89)	56 (2.42)	0.315	58 (2.61)	64 (2.88)	0.582
Peptic ulcer disease	57 (0.63)	12 (0.52)	15 (0.65)	0.563	10 (0.45)	20 (0.90)	0.067
Hemiplegia or paraplegia	33 (0.36)	5 (0.22)	9 (0.39)	0.423	7 (0.32)	12 (0.54)	0.250
Malignancy^3^	816 (8.99)	196 (8.46)	220 (9.50)	0.217	170 (7.65)	230 (10.36)	0.002
AIDS or HIV	0	0	0	-	0	0	-
**CHA**_**2**_**DS**_**2**_**–VASc score**	2.99 (1.56)	3.09 (1.43)	2.73 (1.64)	<0.001	3.29 (1.51)	2.85 (1.61)	<0.001
**HAS-BLED score**^**4**^	1.73 (0.88)	1.80 (0.73)	1.54 (0.94)	<0.001	1.96 (0.82)	1.63 (0.95)	<0.001
**Concurrent medication use interacting with warfarin:** n (%)							
Other anticoagulants	123 (1.36)	34 (1.47)	38 (1.64)	0.635	25 (1.13)	26 (1.17)	0.888
Antiplatelets	836 (9.21)	184 (7.94)	248 (10.71)	0.001	194 (8.73)	210 (9.46)	0.404
NSAIDs	766 (8.44)	253 (10.92)	197 (8.51)	0.006	183 (8.24)	133 (5.99)	0.004
Antibiotics	1726 (19.02)	496 (21.42)	432 (18.65)	0.019	471 (21.21)	327 (14.72)	<0.001
Antifungals	169 (1.86)	38 (1.64)	48 (2.07)	0.276	34 (1.53)	49 (2.21)	0.097
Antitubercular agents	1 (0.01)	1 (0.04)	0 (0)	1.000#	0	0	-
Cardiac drugs	1706 (18.80)	571 (24.65)	323 (13.95)	<0.001	559 (25.17)	253 (11.39)	<0.001
Antilipemic drugs	81 (0.89)	16 (0.69)	13 (0.56)	0.576	31 (1.40)	21 (0.95)	0.163
Antidepressants	1059 (11.67)	263 (11.36)	276 (11.92)	0.551	284 (12.79)	236 (10.63)	0.025
Other CNS drugs	52 (0.57)	13 (0.56)	15 (0.65)	0.705	15 (0.68)	9 (0.41)	0.219
GI drugs	1836 (20.23)	499 (21.55)	403 (17.40)	<0.001	538 (24.22)	396 (17.83)	<0.001
Other drug^5^	255 (2.81)	58 (2.50)	28 (1.21)	0.001	111 (5.00)	58 (2.61)	<0.001
**Laboratory information**: mean (SD)							
Serum creatinine, mg/dl	1.18 (0.78)	1.18 (0.81)	1.24 (0.88)	0.077	1.14 (0.57)	1.17 (0.82)	0.232
INR	1.49 (0.75)	1.60 (0.85)	1.36 (0.67)	<0.001	1.62 (0.79)	1.28 (0.53)	<0.001
Albumin, g/dl	3.85 (0.70)	3.91 (0.65)	3.89 (0.65)	0.553	3.82 (0.71)	3.79 (0.78)	0.300
Hemoglobin, g/dl	13.74 (2.21)	14.00 (2.14)	13.86 (2.14)	0.065	13.52 (2.30)	13.60 (2.22)	0.345

SD = standard deviation; TIA = transient ischemic attack; AIDS or HIV = acquired immune deficiency syndrome or human immunodeficiency virus infection; NSAIDs = non-steroidal anti-inflammatory drugs; CNS drugs = central nervous system drugs; INR = international normalized ratio.

^1^Median follow-up: 652 days (interquartile range: 299 to 1068)

^2^Median follow-up: 628 days (interquartile range: 293 to 1036)

^3^Any malignancy, including lymphoma and leukemia, except malignant neoplasm of skin

^4^ No data on labile INR to calculate the HAS-BLED score

^5^ Other drug included tramadol

# Fisher’s exact test

The derivation cohort (KPCO-I) included 4632 patients with a median follow-up of 652 days, while the validation cohort (KPCO-II) included 4442 patients with a median follow-up of 628 days (**Table 2**). In the KPCO-I cohort, warfarin users were significantly older and had higher proportions of patients with congestive heart failure, hypertension, renal disease, prior major bleeding, anemia, and alcohol abuse than non-users (all p < 0.05). The CHA_2_DS_2_-VASc (mean 3.09 versus 2.73) and HAS-BLED (mean 1.80 versus 1.54) scores were higher in warfarin users. A higher proportion of warfarin users had purchased concurrently an NSAID, antibiotic, cardiac drug, GI drug, and other drug (tramadol) than non-users. However, a lower percentage of antiplatelet use was observed in warfarin users compared with non-users (p = 0.001). Similar characteristics and comparison between warfarin users and non-users were found in the KPCO-II cohort (**Table 2**). **S1 Table** presents the comparison between warfarin users and non-users in the whole cohort (i.e., KPCO-I combined with KPCO-II), with similar results to findings as those from the KPCO-I cohort alone.

Twenty-eight patients (12 and 16 in the KPCO-I and KPCO- II cohorts, respectively) had a stroke and major bleeding outcome on the same date; thus, their time to first event could not be identified. Because of the low frequency, these patients were randomly allocated into either stroke with no major bleeding (n = 14) or major bleeding with no stroke (n = 14). Therefore, in the combined cohort there were 278 strokes (3.06%), 453 major bleedings (4.99%) and 2186 deaths (24.09%) occurred during follow-up. Of these, 136 strokes (2.94%), 280 major bleedings (6.04%) and 1194 deaths (25.78%) occurred in the KPCO-I cohort. In both the KPCO-I and KPCO- II cohorts, the rates of major bleeding and death, but not stroke, differed between warfarin users and non-users (**Table 3)**. Also, as shown in **S2 Fig,** there was a significant difference in all-cause mortality (log-rank p-value = 0.001) between the KPCO-I cohort and KPCO-II cohort.

**Table 3:** Outcomes until study outcome end date between warfarin users and non-users in KPCO-I and KPCO-II cohorts.

Outcomes	Total participants (n = 9074)	KPCO-I (n = 4632)			KPCO-II (n = 4442)		
		Warfarin users (n = 2316)	Warfarin non-users (n = 2316)	P-value	Warfarin users (n = 2221)	Warfarin non-users (n = 2221)	P-value
Stroke, n (%)	278 (3.06)	65 (2.81)	71 (3.07)	0.602	71 (3.20)	71 (3.20)	1.000
Major bleeding, n (%)	453 (4.99)	181 (7.82)	99 (4.27)	<0.001	106 (4.77)	67 (3.02)	0.003
Death, n (%)	2186 (24.09)	442 (19.08)	752 (32.47)	<0.001	355 (15.98)	637 (28.68)	<0.001

Significant trends for increasing stroke and major bleeding rates with higher CHA_2_DS_2_-VASc and HAS-BLED scores were found (p < 0.001) for both the KPCO-I and KPCO-II cohorts **(S2 Table)**.

### PLR Model

The PLR model included age, female sex, warfarin use, CHF, other cerebrovascular disease, hypertension, diabetes, prior major bleeding, prior stroke, renal disease, and concurrent use of antibiotics, antiplatelets, and GI drugs (**Table 4)**. Warfarin use was not associated with stroke (OR = 0.94, 95% CI: 0.66–1.34) but was associated with increased risk of major bleeding (OR = 1.71, 95% CI: 1.32–2.22). All other predictors in the model were associated with an increased risk of outcomes, except hypertension (OR = 0.88, 95% CI: 0.56–1.39) and antibiotic use (OR = 0.98, 95% CI: 0.64–1.51) for stroke, and female sex (OR = 0.73, 95% CI: 0.56–0.94), hypertension (OR = 0.94, 95% CI: 0.66–1.33), and prior stroke (OR = 0.73, 95% CI: 0.40–1.34) for major bleeding.

**Table 4:** Results of the original PLR model and bootstrap analyses for stroke and major bleeding in KPCO-I cohort.

Predictors	Stroke vs. neither event (OR with 95%CI, p-value)		Major bleeding vs. neither event (OR with 95%CI, p-value)	
	Original model	Bootstrap model	Original model	Bootstrap model
Intercept: coefficientβ, p-value	-3.76, <0.001	-3.91, <0.001	-4.21, < 0.001	-4.09, <0.001
Age^1^: years	1.02 (1.00–1.04), 0.013	1.02 (1.01–1.04), 0.010	1.02 (1.01–1.03), 0.001	1.02 (1.01–1.03), 0.001
Female	1.51 (1.06–2.13), 0.025	1.53 (1.05–2.22), 0.024	0.73 (0.56–0.94), 0.015	0.74 (0.56–0.97), 0.028
Warfarin	0.94 (0.66–1.34),0.711	0.97 (0.69–1.43), 0.789	1.71 (1.32–2.22), < 0.001	1.89 (1.46–2.44), <0.001
Other cerebrovascular disease	4.76 (2.42–9.37), <0.001	4.85 (2.34–10.03), <0.001	1.36 (0.60–3.15), 0.469	1.33 (0.39–4.47), 0.338
Congestive heart failure	1.25 (0.71–2.22), 0.434	1.30 (0.68–2.43), 0.427	1.59 (1.14–2.23), 0.007	1.59 (1.14–2.24), 0.008
Hypertension	0.88 (0.56–1.39), 0.587	0.81 (0.54–1.18), 0.326	0.94 (0.66–1.33), 0.712	0.92 (0.64–1.31), 0.695
Diabetes	1.13 (0.72–1.79), 0.598	1.15 (0.70–1.90), 0.412	1.21 (0.89–1.65), 0.233	1.25 (0.89–1.74), 0.177
Prior major bleeding	1.12 (0.50–2.52), 0.782	1.06 (0.42–2.70), 0.783	1.49 (0.87–2.54), 0.147	1.48 (0.84–2.62), 0.149
Prior stroke	2.04 (1.16–3.57), 0.013	2.08 (1.14–3.78), 0.014	0.73 (0.40–1.34), 0.313	0.70 (0.38–1.31), 0.274
Renal disease	1.35 (0.74–2.45), 0.329	1.40 (0.75–2.61), 0.273	1.51 (1.01–2.30), 0.046	1.51 (0.98–2.32), 0.050
Concurrent use of antibiotics	0.98 (0.64–1.51), 0.916	0.97 (0.62–1.53), 0.803	1.81 (1.39–2.37), < 0.001	1.81 (1.38–2.40), <0.001
Concurrent use of antiplatelets	1.71 (1.05–2.77), 0.030	1.67 (1.01–2.76), 0.045	1.57 (1.09–2.27), 0.017	1.57 (1.06–2.33), 0.018
Concurrent use of gastrointestinal medications	1.19 (0.75–1.88), 0.459	1.18 (0.74–1.89), 0.398	1.77 (1.35–2.33), < 0.001	1.79 (1.34–2.39), <0.001

PLR = polytomous logistic regression; OR = odds ratio; CI = confidence interval

^1^ Used as per one-year change

### Cox Model

The all-cause mortality model included age, warfarin, anemia, other cerebrovascular disease, CHF, diabetes, hypertension, prior major bleeding, malignancy, and concurrent use of antifungals and antidepressants (**Table 5**). Warfarin use was associated with a decreased risk of death (HR = 0.55, 95% CI: 0.49–0.62). All other predictors were associated with increased risk of death except hypertension (HR = 0.76, 95% CI: 0.66–0.85).

**Table 5:** Results of the Cox model for death in the KPCO-I cohort.

Predictors	All-cause death (n = 1194)			
	Original model		Bootstrap model	
	HR (95% CI)	P-value	HR (95% CI)	P-value
Age^1^: years	1.06 (1.06–1.07)	<0.001	1.06 (1.06–1.07)	<0.001
Warfarin	0.55 (0.49–0.62)	< 0.001	0.52 (0.47–0.59)	<0.001
Anemia	1.91 (1.61–2.26)	< 0.001	1.91 (1.58–2.30)	<0.001
Other cerebrovascular disease	1.69 (1.22–2.35)	0.001	1.73 (1.22–2.48)	0.002
Congestive heart failure	1.50 (1.29–1.75)	< 0.001	1.49 (1.28–1.76)	<0.001
Diabetes	1.49 (1.29–1.70)	< 0.001	1.53 (1.32–1.76)	<0.001
Hypertension	0.76 (0.66–0.85)	< 0.001	0.76 (0.67–0.86)	<0.001
Prior major bleeding	1.37 (1.07–1.76)	0.012	1.39 (1.09–1.76)	0.007
Malignancy^2^	1.87 (1.59–2.19)	<0.001	1.86 (1.57–2.22)	<0.001
Concurrent use of antifungals	1.56 (1.11–2.17)	0.009	1.56 (1.14–2.13)	0.006
Concurrent use of antidepressants	1.22 (1.04–1.45)	0.013	1.19 (1.03–1.39)	0.015

HR = hazard ratio; CI = confidence interval

^1^ Used as per one-year change

^2^ Any malignancy, including lymphoma and leukemia, except malignant neoplasm of skin

### Sensitivity Analyses

When warfarin use was treated as a time-dependent covariate, similar associations between warfarin and outcomes were found as in the PLR model for stroke and major bleeding and the Cox model for all-cause mortality (**S3 Table**). Results from the competing risk sensitivity analysis for stroke and major bleeding identified similar coefficients for all the predictors included in the PLR model, indicating the robustness of the prediction model (**Table 6)**.

**Table 6:** Sensitivity analysis results from competing risk analysis for stroke and bleeding based on survival analysis in KPCO-I cohort.

Predictors	All-cause death as a competing risk^1^	
	Stroke (n = 136) vs. no stroke (SHR with 95% CI, p-value)	Major bleeding (n = 280) vs. no major bleeding (SHR with 95% CI, p-value)
Age^2^: years	1.01 (1.00–1.03), 0.043	1.01 (1.00–1.03), 0.028
Female	1.56 (1.11–2.22), 0.012	0.75 (0.59–0.96), 0.023
Warfarin	0.94 (0.66–1.36), 0.759	1.84 (1.43–2.36), <0.001
Other cerebrovascular disease	4.31 (2.27–8.20), <0.001	1.21 (0.57–2.58), 0.624
Congestive heart failure	1.35 (0.76–2.38), 0.296	1.52 (1.10–2.11), 0.011
Hypertension	0.92 (0.58–1.44), 0.705	0.93 (0.68–1.29), 0.674
Diabetes	1.07 (0.68–1.69), 0.763	1.18 (0.88–1.59), 0.261
Prior major bleeding	1.06 (0.50–2.26), 0.885	1.40 (0.87–2.27), 0.166
Prior stroke	1.97 (1.14–3.42), 0.015	0.71 (0.39–1.29), 0.259
Renal disease	1.20 (0.67–2.16), 0.546	1.37 (0.93–2.01), 0.108
Concurrent use of antibiotics	0.90 (0.59–1.38), 0.629	1.70 (1.31–2.21), <0.001
Concurrent use of antiplatelets	1.65 (1.02–2.68), 0.042	1.47 (1.02–2.11), 0.037
Concurrent use of gastrointestinal medications	1.25 (0.79–1.95), 0.340	1.75 (1.34–2.28), <0.001

SHR = subdistribution hazard ratio; CI = confidence interval

^1^ The Fine and Gray proportional subdistribution hazards model was used

^2^ Used as per one-year change

### Model performance and validation

The prediction models had a good fit to the data in the derivation cohort (p > 0.05) (**Table 7)**. The discrimination of the models (AUC = 0.71 and 0.72 for stroke and major bleeding, respectively, and C index = 0.75 for all-cause mortality) were acceptable. The overall calibration of the PLR model (**S3 Fig and S4 Fig**) and the Cox model (**S5 Fig**) was satisfactory. Bootstrap analyses for the PLR model and the Cox model yielded the same predictors and similar coefficients as the original models, indicating internal model validation (**Tables 4 and 5**). Findings from 10-fold cross-validation also produced similar AUCs to the original PLR model: 0.69 for stroke and 0.71 for major bleeding (**Table 7**). For external validation in the KPCO-II cohort, the models’ intercepts and the regression coefficients were updated (**S4 Table**). Results of the model goodness-of-fit test (**Table 7**), discrimination (**Table 7**) and calibration (**S6, S7 and S8 Figs** supported external validation for the PLR and Cox models.

**Table 7:** Model performance of PLR model for stroke and major bleeding and Cox model for death in KPCO-I and KPCO-II cohorts.

Model performance	KPCO-I (n = 4632)			KPCO-II (n = 4442)		
	PLR model^3^		Cox model	PLR model		Cox model
	Stroke vs. neither event	Major bleeding vs. neither event	Death vs. survival	Stroke vs. neither event	Major bleeding vs. neither event	Death vs. survival
Goodness-of-fit test statistics (p-value)^1^	8.61 (0.377)	11.08 (0.197)	14.34 (0.114)	10.32 (0.243)	7.30 (0.505)	15.01 (0.093)
Discrimination (95% CI)^2^	0.71 (0.65–0.75)	0.72 (0.68–0.75)	0.75 (0.73–0.76)	0.65 (0.60–0.69)	0.66 (0.62–0.70)	0.76 (0.74–0.77)

PLR = polytomous logistic regression

^1^ Hosmer-Lemeshow test used for the PLR model, Groennesby and Borgan test used for the Cox model

^2^ Area under the receiver operating characteristic curves (AUC) used for the PLR model, Harrell's C index used for the Cox model

^3^ AUC from 10-fold cross-validation for stroke vs. neither event: 0.69 (0.66–0.71), for major bleeding vs. neither event: 0.71 (0.69–0.72)

## Discussion

In this study of patients diagnosed with AF who were and were not initiated on warfarin therapy, we present a new methodology to predict individual combined benefit and harm outcomes of warfarin therapy. We utilized a PLR model to predict the individual benefit and harm outcomes due to its simplicity and flexibility, especially in predictor selection [22,23]. The PLR modelling can incorporate individual baseline characteristics of patients to estimate individual probabilities of the combined benefit and harm outcomes. Compared with the decision tree model which is another commonly-used method for prediction building, the PLR models have shown greater discrimination and predictive accuracy [44–49].

We found that warfarin use, age, female sex, CHF, other cerebrovascular disease, hypertension, diabetes, prior major bleeding, prior stroke, renal disease, and concurrent use of antibiotics, antiplatelets, and GI drugs were included in the PLR model for stroke and major bleeding. Our model performance was acceptable and robust. Using the predictors we identified, the estimated probabilities of the potential outcomes can be computed. For example, if an 82 year-old woman taking warfarin had CHF, diabetes, renal disease and prior major bleeding, and used GI medications concurrently with warfarin, then her log(stroke/neither event) would be -0.85, and log(major bleeding/neither event) would be -0.33, respectively. Subsequently, her estimated 3-year probability of stroke would be: $\frac{e^{-0.85}}{1+e^{-0.85}+e^{-0.33}}$ = 19.9%, her probability of major bleeding would be: $\frac{e^{-0.33}}{1+e^{-0.85}+e^{-0.33}}$ = 33.6%, and her probability of neither event would be: $\frac{1}{1+e^{-0.85}+e^{-0.33}}$ = 46.5% [23]. By contrast if she did not start warfarin therapy but all other factors were the same, her estimated probability of stroke, major bleeding and neither event would be 24.3%, 22.5% and 53.2%, respectively. Likewise, her estimated 3-year probability of all-cause mortality with and without warfarin therapy initiation would be 6.9% and 24.4% respectively, using the Cox model.

In our prediction models, warfarin was associated with an increased risk of major bleeding and decreased risk of death, which is in accordance with previous findings [50,51]. However, we did not identify an association between warfarin use and decreased risk of stroke. A possible explanation for this unexpected observation might include lack of INR control measures, such as time in therapeutic range (TTR), in our prediction models. Prior research indicates that the full benefit of stroke risk reduction may require an individual TTR of at least 70% in warfarin users [52]. However, individual TTR results for patients in our cohorts could not be included in the models since warfarin non-users were unmeasured on this factor. Another possible explanation relates to our use of ICD-9-CM codes alone to identify stroke and bleeding outcomes without confirmatory chart review. The positive predictive values of ICD-9-CM codes for bleeding have been shown to be higher than those for stroke [53,54]; thus, the use of ICD-9-CM codes alone may have provided a high rate of stroke false positives. In addition, a stroke history may have increased the likelihood that a given patient received warfarin to prevent further stroke risk and concurrently increased the likelihood that false positive stroke ICD-9-CM codes were identified during administrative data acquisition.

The CHADS_2_/CHA_2_DS_2_-VASc, and HAS-BLED scores are used worldwide in patients with AF to stratify the risk of stroke and major bleeding, respectively. However, these risk-stratification tools cannot provide the individual combined benefit and harm assessments needed by patients and physicians at inception of warfarin therapy or when concerns arise during ongoing use. Moreover, concerns have been expressed about their scoring algorithms and poor discrimination [55–59]. For instance, in one study compared with their peers with a CHA_2_DS_2_-VASc score of 0 and 1 for men and women, respectively, the unequal risk of stroke for the additional risk factors resulted in different weighting in the scoring algorithm. This corresponded to a HR of from 1.68 with vascular disease to 3.09 with an age of 65–74 years for men and a HR of from 1.71 with hypertension to 3.03 with an age of 65–74 years for women [57]. Therefore given the potential different weighting for individual components of the scores as well as more detailed information provided by the individual components, we used individual risk factors, rather than gross risk scores, in our model construction.

Other studies have used the ‘net benefit’ approach of considering stroke and major bleeding outcomes simultaneously [19–21]. Unfortunately, GI bleeding risk was not considered, and the weighting factor reflecting the importance of ICH was chosen subjectively and arbitrarily in these studies. Additionally, while some studies have combined stroke and bleeding risk-stratification scores to calculate overall clinical outcome risks including stroke and major bleeding [60,61], they did not improve prediction of stroke and major bleeding beyond the individual stroke (CHADS_2_, CHA_2_DS_2_-VASc) or bleeding scores (HAS-BLED) [62]. In contrast, our study may provide insights into using a new methodology to take into account individual benefit-harm outcomes with warfarin therapy. Our PLR model calculates the specific probabilities of stroke and major bleeding at the same time, which may be more practical and acceptable in real-world clinical practice compared with using separate stroke and bleeding risk-stratification scores. Moreover, because our model produces individualized risk estimates for each patient based on various characteristics, it offers more personalized and detailed information for patients with AF rather than the population-level estimates associated with CHADS_2_, CHA_2_DS_2_-VASc, and HAS-BLED scores [23]. Thus the PLR model may better facilitate patient-physician shared decision-making with regard to warfarin therapy initiation.

In our study, an unexpected inverse association between comorbid hypertension and stroke, major bleeding, and all-cause death was observed. During the model construction, we used either the ICD-9-CM codes or the antihypertensive drug surrogates including angiotensin-converting enzyme inhibitors, angiotensin II receptor blockers, thiazides, beta-blockers, calcium channel blockers, and other antihypertensive purchases, to identify hypertension comorbidity (**S5 Table**). Additionally, we ran two *post-hoc* sensitivity analyses using different methods to imply hypertension diagnosis: ICD-9-CM codes only, and both ICD-9-CM codes and antihypertensive drug purchases. These two methods yielded the same predictors included in the PLR and Cox model with extremely similar coefficients (**S6 Table**). Moreover, removing hypertension from the model entirely also yielded similar results (**S7 Table for the PLR model; S8 Table for the Cox model**). Therefore, the unexpected relationship between hypertension and outcomes requires further exploration.

The strengths of our study include the use of a large sample of patients with AF to construct and validate the prediction model. Moreover, model building, assessment, and validation included rigorous and detailed statistical analyses. Another strength is the efforts in controlling bias in study design and data analyses to preclude misleading predictors from being included into the models. Nevertheless, our study also has several limitations. The majority of the data used in this study were from ICD-9-CM codes only without confirmatory chart review of the diagnosis. Thus data accuracy for baseline comorbidities may be less than optimal. Likewise, the incidence rates of stroke and major bleeding may be over- or underestimated. This could lead to false positive/negative values and weaken the findings based on the data. Additionally, we intended to predict four outcome quadrants (**Table 1**). However, the number of patients experiencing simultaneous stroke and major bleeding (n = 28) was insufficient for model construction. Another limitation is lack of data from contemporary non-KPCO cohorts for model validation; thereby, potentially limiting the generalizability of the prediction model [27].

## Conclusions

In this study, we introduce a new methodology for predicting individual combined benefit and harm outcomes associated with warfarin therapy for patients with AF. Should this approach be validated in other patient populations, it has potential advantages over existing risk stratification approaches as a patient-physician aid for shared decision-making.

## Supporting Information

S1 Fig Flow diagram of selecting patients for analyses. (DOCX)

S2 Fig Kaplan-Meier survival curves for death in the derivation and validation cohorts. (DOCX)

S3 Fig Calibration curve in the PLR model for stroke in the derivation cohort. (DOCX)

S4 Fig Calibration curve in the PLR model for major bleeding in the derivation cohort. (DOCX)

S5 Fig Calibration curve in the Cox model for death in the derivation cohort. (DOCX)

S6 Fig Calibration curve in the PLR model for stroke in the validation cohort. (DOCX)

S7 Fig Calibration curve in the PLR model for major bleeding in the validation cohort. (DOCX)

S8 Fig Calibration curve in the Cox model for death in the validation cohort. (DOCX)

S1 Table Characteristics of study patients stratified by taking versus not taking warfarin for the whole cohort. (DOCX)

S2 Table Rates of stroke and major bleeding in the KPCO cohorts stratified by CHA_2_DS_2_-VASc and HAS-BLED scores. (DOCX)

S3 Table Sensitivity analysis results from multivariable model to assess time-varying effect of warfarin on stroke, major bleeding and death. (DOCX)

S4 Table Updates of the models’ intercepts and the regression coefficients for external validation in the KPCO-Ⅱ cohort. (DOCX)

S5 Table Hypertensive drugs as surrogates for hypertension. (DOCX)

S6 Table Results for effect of hypertension in the PLR and Cox model using different data on hypertension in the KPCO-I cohort. (DOCX)

S7 Table Sensitivity analysis leaving hypertension out of the PLR model for stroke and major bleeding in the KPCO-I cohort. (DOCX)

S8 Table Sensitivity analysis leaving hypertension out of the Cox model for death in the KPCO-I cohort. (DOCX)
