# Supplementary material for: Can We Predict Individual Combined Benefit and Harm of Therapy? Warfarin Therapy for Atrial Fibrillation as a Test Case
Source: PLoS One. 2016 Aug 11;11(8):e0160713. doi: 10.1371/journal.pone.0160713 (PMC4981352; doi:10.1371/journal.pone.0160713)
Supplement: S1 Fig — (DOCX) [file pone.0160713.s001.docx]

**S1 Fig. Flow diagram of selecting patients for analyses**

Patients with first AF diagnoses from 1/1/2005 to 12/31/2012 and no AF diagnosis in the previous 180 days

(n=22,438)

Ineligible patients excluded (n=8,907):

1. KPCO membership less than 180 days prior to AF diagnoses (n= 5,779)
2. Age <18 years (n=27)
3. Warfarin-users with a purchase during the 180 days prior to AF diagnosis or a supply from a warfarin purchase that overlaps into the 180 days (n=3,101)

Eligible patients to enter cohort

(n=13,531)

Patients excluded (n=2,003):

1. Warfarin-users: time range from AF diagnosis to inception of warfarin exceeded 180 days (n=401)
2. Warfarin-users unmatched (n=9)
3. Non-users: failed to match warfarin-users’ time distribution (n=1,490)
4. Non-users: died prior to index date **(**n=103)

Eligible patients in derivation set from 1/1/2005 to 12/31/2008

(n=6,635)

Patients excluded (n=2,454):

1. Warfarin-users: time range from AF diagnosis to inception of warfarin exceeded 180 days (n=343)
2. Warfarin-users unmatched (n=13)
3. Non-users: failed to match warfarin-users’ time distribution (n=1,962)
4. Non-users: died prior to index date (n=136)

Eligible patients in validation set from 1/1/2009 to 12/31/2012

(n=6,896)

Eligible patients for analyses in validation set from 1/1/2009 to 12/31/2012

(n=4,442)

Eligible patients for analyses in derivation set from 1/1/2005 to 12/31/2008

(n=4,632)
