# Supplementary figures and images for: Can We Predict Individual Combined Benefit and Harm of Therapy? Warfarin Therapy for Atrial Fibrillation as a Test Case
Source: PLoS One. 2016 Aug 11;11(8):e0160713. doi: 10.1371/journal.pone.0160713 (PMC4981352; doi:10.1371/journal.pone.0160713)

**S2 Fig. Kaplan-Meier survival curves for death in the derivation and validation cohorts**

**
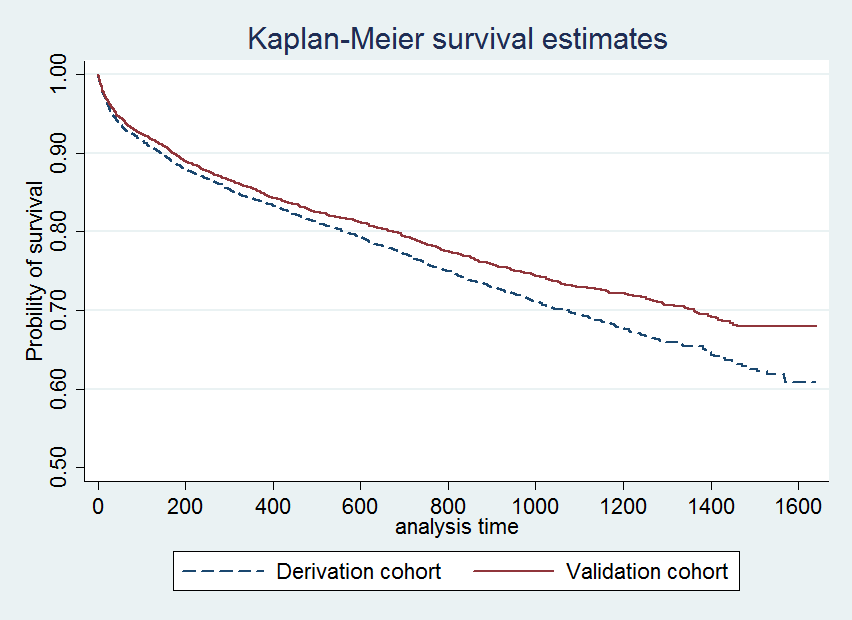
**

Supplement: S2 Fig — (DOCX) [file pone.0160713.s002.docx]

**S3 Fig. Calibration curve in the PLR model for stroke in the derivation cohort**


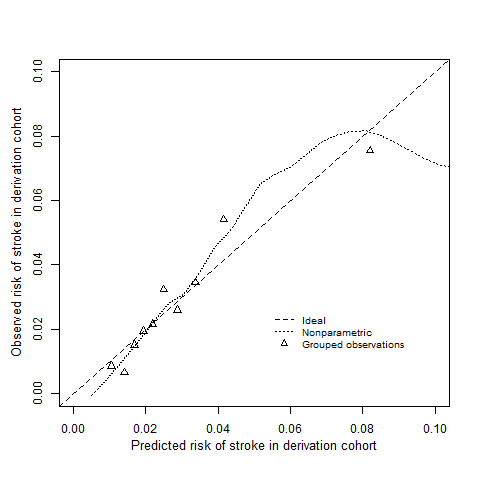

Supplement: S3 Fig — (DOCX) [file pone.0160713.s003.docx]

**S4 Fig. Calibration curve in the PLR model for major bleeding in the derivation cohort**


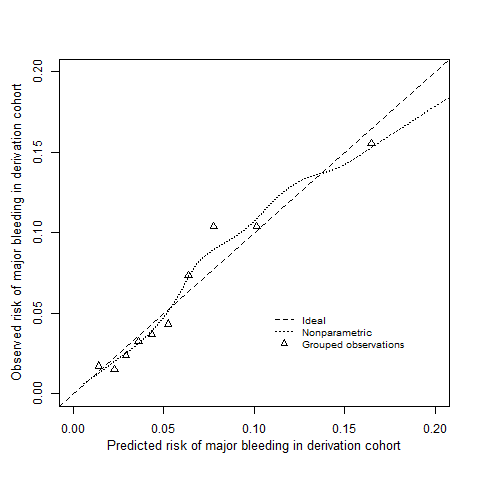

Supplement: S4 Fig — (DOCX) [file pone.0160713.s004.docx]

**S5 Fig. Calibration curve in the Cox model for death in the derivation cohort**


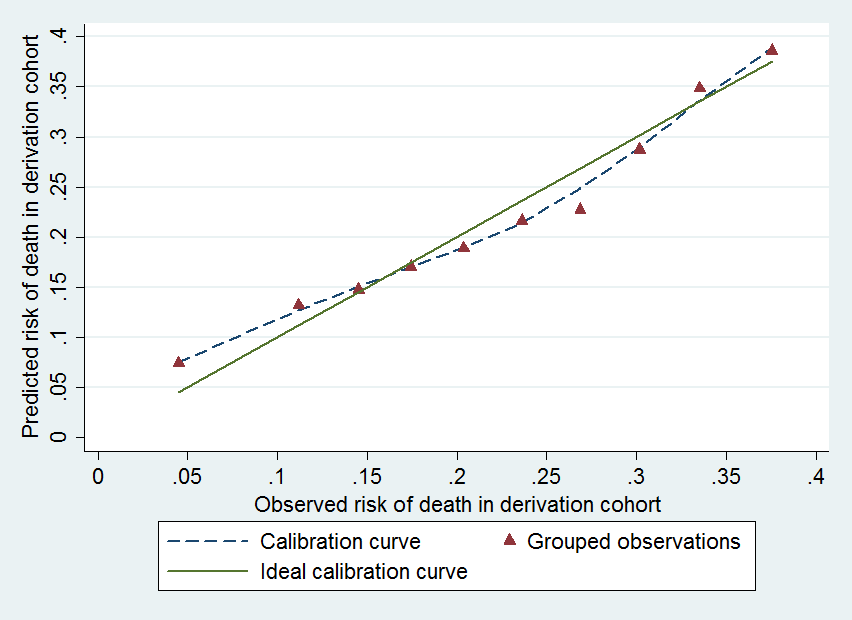

Supplement: S5 Fig — (DOCX) [file pone.0160713.s005.docx]

**S6 Fig. Calibration curve in the PLR model for stroke in the validation cohort**


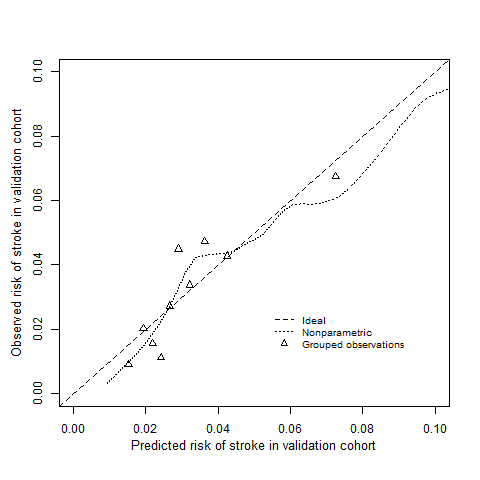

Supplement: S6 Fig — (DOCX) [file pone.0160713.s006.docx]

**S7 Fig. Calibration curve in the PLR model for major bleeding in the validation cohort**


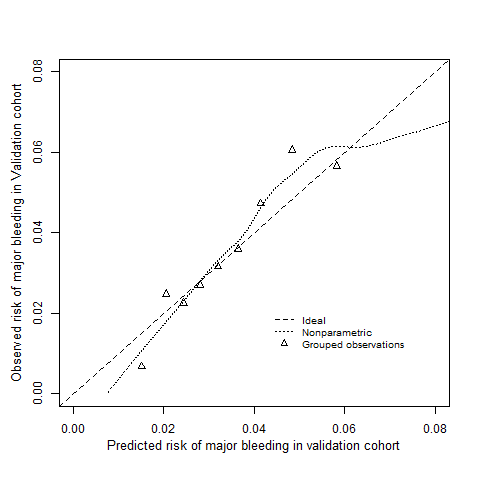

Supplement: S7 Fig — (DOCX) [file pone.0160713.s007.docx]

**S8 Fig. Calibration curve in the Cox model for death in the validation cohort**


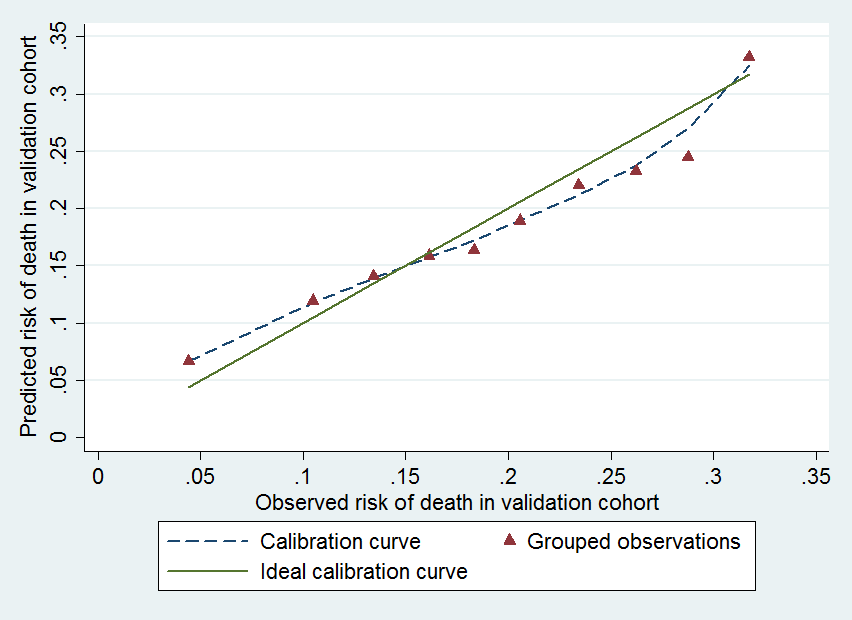

Supplement: S8 Fig — (DOCX) [file pone.0160713.s008.docx]
