# Supplementary material for: Can We Predict Individual Combined Benefit and Harm of Therapy? Warfarin Therapy for Atrial Fibrillation as a Test Case
Source: PLoS One. 2016 Aug 11;11(8):e0160713. doi: 10.1371/journal.pone.0160713 (PMC4981352; doi:10.1371/journal.pone.0160713)
Supplement: S1 Table — (DOCX) [file pone.0160713.s009.docx]

**S1Table. Characteristics of study patients stratified by taking versus not taking warfarin for the whole cohort**

| **Baseline Characteristics** | **KPCO cohort** | | | |
| --- | --- | --- | --- | --- |
|  | Total participants (n=9074) | Warfarin-users  (n=4537) | Warfarin non-users  (n=4537) | P-value |
| **Age:** mean (SD), years | 71.7 (13.00) | 72.6 (10.69) | 70.9 (14.92) | <0.001 |
| **Female**: n (%) | 4199 (46.28) | 2033 (44.81) | 2166 (47.74) | 0.005 |
| **Comorbidities:** n (%) | | | | |
| Congestive heart failure | 1064 (11.73) | 611 (13.47) | 453 (9.98) | <0.001 |
| Hypertension | 7132 (78.60) | 3981 (87.75) | 3151 (69.45) | <0.001 |
| Diabetes | 1759 (19.39) | 937 (20.65) | 822 (18.12) | 0.002 |
| Prior stroke/TIA | 539 (5.94) | 319 (7.03) | 220 (4.85) | <0.001 |
| Myocardial infarction | 516 (5.69) | 276 (6.08) | 240 (5.29) | 0.103 |
| Peripheral vascular disease | 615 (6.78) | 321 (7.08) | 294 (6.48) | 0.260 |
| Renal disease | 1146 (12.63) | 580 (12.78) | 566 (12.48) | 0.658 |
| Liver disease | 20 (0.22) | 5 (0.11) | 15 (0.33) | 0.041# |
| Prior major bleeding | 260 (2.87) | 116 (2.56) | 144 (3.17) | 0.078 |
| Anemia | 657 (7.24) | 269 (5.93) | 388 (8.55) | <0.001 |
| Alcohol abuse | 119 (1.31) | 39 (0.86) | 80 (1.76) | <0.001 |
| Other cerebrovascular disease | 194 (2.14) | 96 (2.12) | 98 (2.16) | 0.885 |
| Dementia | 21 (0.23) | 3 (0.07) | 18 (0.40) | 0.002# |
| Chronic pulmonary disease | 468 (5.16) | 268 (5.91) | 200 (4.41) | 0.001 |
| Rheumatic disease | 245 (2.70) | 125 (2.76) | 120 (2.64) | 0.746 |
| Peptic ulcer disease | 57 (0.63) | 22 (0.48) | 35 (0.77) | 0.084 |
| Hemiplegia or paraplegia | 33 (0.36) | 12 (0.26) | 21 (0.46) | 0.117 |
| Malignancy^1^ | 816 (8.99) | 366 (8.07) | 450 (9.92) | 0.002 |
| AIDS or HIV | 0 | 0 | 0 | - |
| **CHA_2_DS_2_–VASc score** | 2.99 (1.56) | 3.19 (1.47) | 2.79 (1.63) | <0.001 |
| **HAS-BLED score^2^** | 1.73 (0.88) | 1.88 (0.77) | 1.58 (0.95) | <0.001 |
| **Concurrent medication use interacting with warfarin:** n (%) | | | | |
| Other anticoagulants | 123 (1.36) | 59 (1.30) | 64 (1.41) | 0.650 |
| Antiplatelets | 836 (9.21) | 378 (8.33) | 458 (10.09) | 0.004 |
| NSAIDs | 766 (8.44) | 436 (9.61) | 330 (7.27) | <0.001 |
| Antibiotics | 1726 (19.02) | 967 (21.31) | 759 (16.73) | <0.001 |
| Antifungals | 169 (1.86) | 72 (1.59) | 97 (2.14) | 0.052 |
| Antitubercular agents | 1 (0.01) | 1 (0.02) | 0 (0) | 1.000# |
| Cardiac drugs | 1706 (18.80) | 1130 (24.91) | 576 (12.70) | <0.001 |
| Antilipemic drugs | 81 (0.89) | 47 (1.04) | 34 (0.75) | 0.147 |
| Antidepressants | 1059 (11.67) | 547 (12.06) | 512 (11.28) | 0.253 |
| Other CNS drugs | 52 (0.57) | 28 (0.62) | 24 (0.53) | 0.578 |
| GI drugs | 1836 (20.23) | 1037 (22.86) | 799 (17.61) | <0.001 |
| Other drug | 255 (2.81) | 169 (3.72) | 86 (1.90) | <0.001 |
| **Laboratory information**: mean (SD) | | | | |
| Serum creatinine, mg/dl | 1.18 (0.78) | 1.16 (0.69) | 1.20 (0.85) | 0.028 |
| INR | 1.49 (0.75) | 1.61 (0.82) | 1.32 (0.60) | <0.001 |
| Albumin, g/dl | 3.85 (0.70) | 3.86 (0.68) | 3.84 (0.72) | 0.288 |
| Hemoglobin, g/dl | 13.74 (2.21) | 13.75 (2.24) | 13.73 (2.18) | 0.705 |

^1^ Any malignancy, including lymphoma and leukemia, except malignant neoplasm of skin;

^2^ No data on labile INR to calculate the HAS-BLED score;

# Fisher’s exact test
