# Supplementary material for: Can We Predict Individual Combined Benefit and Harm of Therapy? Warfarin Therapy for Atrial Fibrillation as a Test Case
Source: PLoS One. 2016 Aug 11;11(8):e0160713. doi: 10.1371/journal.pone.0160713 (PMC4981352; doi:10.1371/journal.pone.0160713)
Supplement: S2 Table — (DOCX) [file pone.0160713.s010.docx]

**S2 Table. Rates of stroke and major bleeding in the KPCO cohorts stratified by CHA_2_DS_2_-VASc and HAS-BLED scores***

|  | **KPCO-I** | **KPCO-II** | **P-value** |
| --- | --- | --- | --- |
| **Stroke stratified by CHA_2_DS_2_–VASc score: event number/total number (%)** | | | |
| 0 | 1/258 (0.39) | 1/216 (0.46) | 0.998# |
| 1 | 16/683 (2.34) | 12/562 (2.14) | 0.806 |
| 2 | 17/894 (1.90) | 19/822 (2.31) | 0.554 |
| 3 | 26/1078 (2.41) | 29/1050 (2.76) | 0.611 |
| 4 | 46/1076 (4.28) | 42/1040 (4.04) | 0.785 |
| 5 | 22/443 (4.97) | 17/494 (3.44) | 0.243 |
| 6 | 7/146 (4.79) | 12/184 (6.52) | 0.504 |
| 7 | 0/43 (0) | 8/57 (14.04) | 0.011# |
| 8 | 0/9 (0) | 2/17 (11.76) | 0.529# |
| 9 | 1/2 (50.00) | - | - |
| Total | 136/4632 (2.94)^1^ | 142/4442 (3.20)^2^ | 0.471 |
| **Major bleeding stratified by HAS-BLED score^3^: n (%)** | | | |
| 0 | 15/427 (3.51) | 6/357 (1.68) | 0.114 |
| 1 | 61/1325 (4.60) | 28/1178 (2.38) | 0.003 |
| 2 | 157/2287 (6.86) | 91/2045 (4.45) | 0.001 |
| 3 | 39/528 (7.39) | 42/759 (5.53) | 0.178 |
| 4 | 8/62 (12.90) | 6/98 (6.12) | 0.139 |
| 5 | 0/3 (0) | 0/5 (0) | - |
| 6 | - | - | - |
| 7 | - | - | - |
| 8 | - | - | - |
| Total | 280/4632 (6.04)^4^ | 173/4442 (3.89)^5^ | <0.001 |

* Patients’ CHA_2_DS_2_–VASc score (minimum to maximum): 0 to 9; HAS-BLED score (minimum to maximum): 0 to 5

^1^P-value for trend < 0.001; ^2^P-value for trend < 0.001; ^3^No data on labile INR to calculate the HAS-BLED score;

^4^P-value for trend < 0.001; ^5^P-value for trend < 0.001; # Fisher’s exact test
