# Supplementary material for: Can We Predict Individual Combined Benefit and Harm of Therapy? Warfarin Therapy for Atrial Fibrillation as a Test Case
Source: PLoS One. 2016 Aug 11;11(8):e0160713. doi: 10.1371/journal.pone.0160713 (PMC4981352; doi:10.1371/journal.pone.0160713)
Supplement: S3 Table — (DOCX) [file pone.0160713.s011.docx]

**S3 Table. Sensitivity analysis results from multivariable model to assess time-varying effect^1^ of warfarin on stroke, major bleeding and death**

| **Predictor** | **Stroke^2^**  (HR with 95% CI, p-value) | **Major bleeding^2^**  (HR with 95% CI, p-value) | **Death^3^**  (HR with 95% CI, p-value) |
| --- | --- | --- | --- |
| Derivation cohort (n=4632) | | | |
| Warfarin | 0.87 (0.57-1.32), 0.442 | 1.70 (1.28-2.26), <0.001 | 0.36 (0.28-0.45), <0.001 |
| Validation cohort (n=4442) | | | |
| Warfarin | 0.89 (0.57-1.40), 0.618 | 1.24 (0.78-1.96), 0.363 | 0.47 (0.37-0.58), <0.001 |

^1^ Three Cox models were used to assess the time-dependent effect of warfarin on stroke, major bleeding and death, respectively;

^2^ Adjusted for age, sex, other cerebrovascular disease, congestive heart failure, hypertension, diabetes, prior major bleeding, prior stroke, renal disease, concurrent use of antibiotics, concurrent use of antiplatelets, and concurrent use of gastrointestinal medications;

^3^ Adjusted for age, anemia, other cerebrovascular disease, congestive heart failure, hypertension, diabetes, prior major bleeding, malignancy, concurrent use of antifungals, and concurrent use of antidepressants
