# Supplementary material for: Can We Predict Individual Combined Benefit and Harm of Therapy? Warfarin Therapy for Atrial Fibrillation as a Test Case
Source: PLoS One. 2016 Aug 11;11(8):e0160713. doi: 10.1371/journal.pone.0160713 (PMC4981352; doi:10.1371/journal.pone.0160713)
Supplement: S4 Table — (DOCX) [file pone.0160713.s012.docx]

**S4 Table. Updates of the models’ intercepts and the regression coefficients for external validation in the KPCO-Ⅱ cohort**

| **Recalibration of model** | **Update of the PLR model** | | **Update of the Cox model** |
| --- | --- | --- | --- |
|  | **Stroke (n=142) vs. neither event** | **Major bleeding (n=173) vs. neither event** | **All-cause death (n=992) vs. survivors** |
| Calibration intercept (95% CI, p-value) | -2.74 (-2.98 to -2.50),  < 0.001 | -3.58 (-3.79 to -3.37),  < 0.001 | Not specified |
| Calibration slope (95% CI, p-value) | 0.70 (0.49 to 0.91),  < 0.001 | 0.66 (0.47 to 0.85),  < 0.001 | 1.02 (0.95-1.09),  < 0.001 |
