# Supplementary material for: Can We Predict Individual Combined Benefit and Harm of Therapy? Warfarin Therapy for Atrial Fibrillation as a Test Case
Source: PLoS One. 2016 Aug 11;11(8):e0160713. doi: 10.1371/journal.pone.0160713 (PMC4981352; doi:10.1371/journal.pone.0160713)
Supplement: S5 Table — (DOCX) [file pone.0160713.s013.docx]

**S5 Table. Hypertensive drugs as surrogates for hypertension**

| **Group** | **Drug list** |
| --- | --- |
| Angiotensin-converting enzyme inhibitors | captopril, benazepril, enalapril, lisinopril, fosinopril, ramipril, guinapril, tranolopril, perinodopril |
| Angiotensin II receptor blockers | candesartan, eprosartan, irbesartan, losartan, olmesartan, telmisartan, valsartan |
| Thiazides | chlorthiazide, benzthiazide, trichlormethiazide, hydrochlorthiazide, hydroflumethiazide, chlorthalidone, indapamide, methylclothiazide, polythiazide, metolazone |
| Beta-blockers | alprenolol, carteolol, levobunolol, mepindolol, nadolol, oxprenolol, penbutolol, pindolol, propranolol, sotalol, timolol, acebutalol, atenolol, betaxolol, bisoprolol, esmolol, metoprolol, nebivolol, carvedilol, celiprolol, labetolol |
| Calcium channel blockers | amlodipine, felodipine, nicardipine, nifedipine, nimodipine, nisoldipine, nitrendipine, lacidipine, verapamil, diltiazem |
| Others | clonidine, methyldopa, hydralazine |
