# Supplementary material for: Can We Predict Individual Combined Benefit and Harm of Therapy? Warfarin Therapy for Atrial Fibrillation as a Test Case
Source: PLoS One. 2016 Aug 11;11(8):e0160713. doi: 10.1371/journal.pone.0160713 (PMC4981352; doi:10.1371/journal.pone.0160713)
Supplement: S6 Table — (DOCX) [file pone.0160713.s014.docx]

**S6 Table. Results for effect of hypertension in the PLR and Cox model using different data on hypertension in the KPCO-I cohort***

| **Effect of hypertension** | **PLR model** (expressed as OR with 95% CI, p-value) | | **Cox model** (expressed as HR with 95% CI, p-value) |
| --- | --- | --- | --- |
|  | **Stroke vs. neither event** | **Major bleeding vs. neither event** | **Death vs. survival** |
| Either ICD-9-CM codes or hypertensive drugs | 0.88 (0.56-1.39), 0.587 | 0.94 (0.66-1.33), 0.712 | 0.76 (0.66-0.85), <0.001 |
| ICD-9-CM Codes only | 0.80 (0.55-1.17), 0.256 | 0.84 (0.64-1.09), 0.195 | 0.75 (0.67-0.85), <0.001 |
| Both ICD-9-CM codes and hypertensive drugs | 0.86 (0.66-1.12), 0.269 | 0.97 (0.67-1.42), 0.893 | 0.76 (0.67-0.86), <0.001 |

*The three approaches produced the same predictors included in the models and extremely similar coefficients; coefficients for the other predictors not shown here.
