# Supplementary material for: Can We Predict Individual Combined Benefit and Harm of Therapy? Warfarin Therapy for Atrial Fibrillation as a Test Case
Source: PLoS One. 2016 Aug 11;11(8):e0160713. doi: 10.1371/journal.pone.0160713 (PMC4981352; doi:10.1371/journal.pone.0160713)
Supplement: S7 Table — (DOCX) [file pone.0160713.s015.docx]

**S7 Table. Sensitivity analysis leaving hypertension out of the PLR model for stroke and major bleeding in the KPCO-I cohort**

| **Predictors** | **Stroke vs. neither event (OR with 95%CI, p-values)^1^** | **Major bleeding vs. neither event (OR with 95%CI, p-values)^2^** |
| --- | --- | --- |
| Intercept: coefficientβ, p-value | -3.89, <0.001 | -4.02, <0.001 |
| Age^3^ | 1.02 (1.00-1.03), 0.015 | 1.02 (1.01-1.03), 0.001 |
| Female | 1.50 (1.05-2.15), 0.027 | 0.73 (0.56-0.94), 0.014 |
| Warfarin | 0.98 (0.69-1.40), 0.931 | 1.87 (1.45-2.42), < 0.001 |
| Other cerebrovascular disease | 4.79 (2.44-9.42), <0.001 | 1.38 (0.60-3.19), 0.454 |
| Congestive heart failure | 0.79 (0.44-1.40), 0.412 | 1.57 (1.12-2.19), 0.008 |
| Diabetes | 1.11 (0.71-1.75), 0.650 | 1.19 (0.88-1.62), 0.261 |
| Prior major bleeding | 1.13 (0.50-2.54), 0.767 | 1.49 (0.87-2.55), 0.144 |
| Prior stroke | 2.02 (1.15-3.54), 0.014 | 0.71 (0.39-1.30), 0.262 |
| Renal disease | 1.34 (0.74-2.43), 0.339 | 1.47 (0.98-2.21), 0.065 |
| Concurrent use of antibiotics | 0.98 (0.63-1.50), 0.912 | 1.81 (1.38-2.36), < 0.001 |
| Concurrent use of antiplatelets | 1.70 (1.05-2.74), 0.032 | 1.58 (1.09-2.27), 0.016 |
| Concurrent use of gastrointestinal medications | 0.83 (0.53-1.31), 0.425 | 1.76 (1.34-2.31), < 0.001 |

^1^ AUC for stroke versus neither event: 0.69 (0.65-0.74); Hosmer-Lemeshow test statistics (p-value): 8.89 (0.352)

^2^ AUC for major bleeding versus neither event: 0.71 (0.68-0.75); Hosmer-Lemeshow test statistics (p-value): 10.02 (0.264)

^3^ Used as per one-year change
