# Supplementary material for: Can We Predict Individual Combined Benefit and Harm of Therapy? Warfarin Therapy for Atrial Fibrillation as a Test Case
Source: PLoS One. 2016 Aug 11;11(8):e0160713. doi: 10.1371/journal.pone.0160713 (PMC4981352; doi:10.1371/journal.pone.0160713)
Supplement: S8 Table — (DOCX) [file pone.0160713.s016.docx]

**S8 Table. Sensitivity analysis leaving hypertension out of the Cox model for death in the KPCO-I cohort**

| **Predictors** | **All-cause death (n=1194)^1^** | |
| --- | --- | --- |
|  | HR (95% CI) | p-value |
| Age^2^ | 1.06 (1.06-1.07) | <0.001 |
| Warfarin | 0.52 (0.46-0.58) | < 0.001 |
| Anemia | 1.85 (1.56-2.19) | < 0.001 |
| Other cerebrovascular disease^3^ | 1.68 (1.21-2.34) | 0.002 |
| Congestive heart failure | 1.46 (1.26-1.71) | < 0.001 |
| Diabetes | 1.41 (1.23-1.62) | < 0.001 |
| Prior major bleeding | 1.37 (1.07-1.75) | 0.014 |
| Malignancy^2^ | 1.82 (1.56-2.14) | <0.001 |
| Concurrent use of antifungals | 1.57 (1.12-2.19) | 0.008 |
| Concurrent use of antidepressants | 1.21 (1.03-1.42) | 0.023 |

^1^ C-index = 0.74 (0.73-0.76); Groennesby and Borgan test statistic (p-value): 14.05 (0.120)

^2^ Used as per one-year change; ^3^ Any malignancy, including lymphoma and leukemia, except malignant neoplasm of skin
